# Regression of cardiac angiosarcoma in a 17-year-old: a percutaneous biopsy effect

**DOI:** 10.1186/s40959-024-00239-9

**Published:** 2024-07-23

**Authors:** Noor Sharrack, Martine Parent, Christopher Lethaby, Ulrich Rosendahl, Alexander R Lyon, Maryum Farooq, Haqeel Jamil, John P. Greenwood, Sven Plein, Ananth Kidambi

**Affiliations:** 1 Leeds Institute of Cardiovascular and Metabolic Medicine, University of Leeds, Leeds, LS2 9JT UK; 2 Yorkshire Regional Centre for Pediatric Oncology and Hematology, Leeds General Infirmary, Leeds, Yorkshire, UK; 3 Department of Cardiac Surgery, Royal Brompton and Harefield Hospitals, London, UK; 4 National Heart and Lung Institute, Imperial College London, London, UK; 5 Cardio-Oncology Service, Royal Brompton Hospital, London, UK; 6 Department of Cardiology, Airedale NHS foundation trust, West Yorkshire, UK; 7 Department of Cardiology, Leeds Teaching Hospitals, Leeds, UK

**Keywords:** Primary cardiac angiosarcoma (PCAS), Cardiovascular Magnetic Resonance (CMR), Percutaneous biopsy, Tumor regression

## Abstract

**Background:**

Cardiac angiosarcoma is a very rare and aggressive primary cardiac tumor associated with poor prognosis. Diagnosis is often delayed due to non-specific symptoms, with most cases involving metastases at the time of diagnosis. We describe a unique case of apparent tumor regression of cardiac angiosarcoma post percutaneous biopsy.

**Case Presentation:**

A young male was admitted with suspected pericarditis. Echocardiogram revealed a pericardial mass. Cardiovascular magnetic resonance (CMR) suggested primary cardiac malignancy. Percutaneous biopsy was inconclusive, with subsequent CMR demonstrating apparent tumor regression. Interval imaging revealed further tumor growth, and surgical biopsy revealed primary cardiac angiosarcoma (PCAS). Causes of tumor regression following percutaneous biopsy are discussed.

**Conclusions:**

Cases of suspected primary cardiac malignancy require careful follow up with serial multimodality imaging. Percutaneous biopsy effects should be considered in cases of tumor regression, and serial imaging should be planned afterwards.

**Supplementary Information:**

The online version contains supplementary material available at 10.1186/s40959-024-00239-9.

## Background

Primary cardiac angiosarcoma (PCAS) is rare and has a very poor prognosis with median survival ranging from 6 to 11 months [1]. In adult autopsy series, the incidence is 0.0001% [2]. Cardiac sarcomas comprise around 95% of primary malignant cardiac tumors, with PCAS and undifferentiated sarcoma being the most common subtypes [3]. PCAS are the most aggressive histotype and account for around a third of all primary cardiac malignant tumors [3]. PCAS mostly occurs in the right atrium (RA) or right ventricle (RV), with invasive growth and metastases that often infiltrate the myocardium, valves, pericardium and coronary arteries.

## Case presentation

A fit and healthy 17-year-old male was admitted to his local hospital with a 10-day history of chest pain and breathlessness. The chest pain was inspiratory, non-radiating, worse on lying flat and relieved by leaning forward. He had no history of fever, night sweats or weight loss. The clinical picture suggested pericarditis. His past medical history was limited to recurrent ear infections as a child. He was not taking any regular medication. He was a college student, normally fit and well, played rugby and martial arts. There was no significant family history of note. Given the clinical history and examination findings, initial differential diagnoses included pericarditis, myocarditis, and pneumonia. First line investigations were ordered to establish the cause of presentation.

**Fig. 1:**
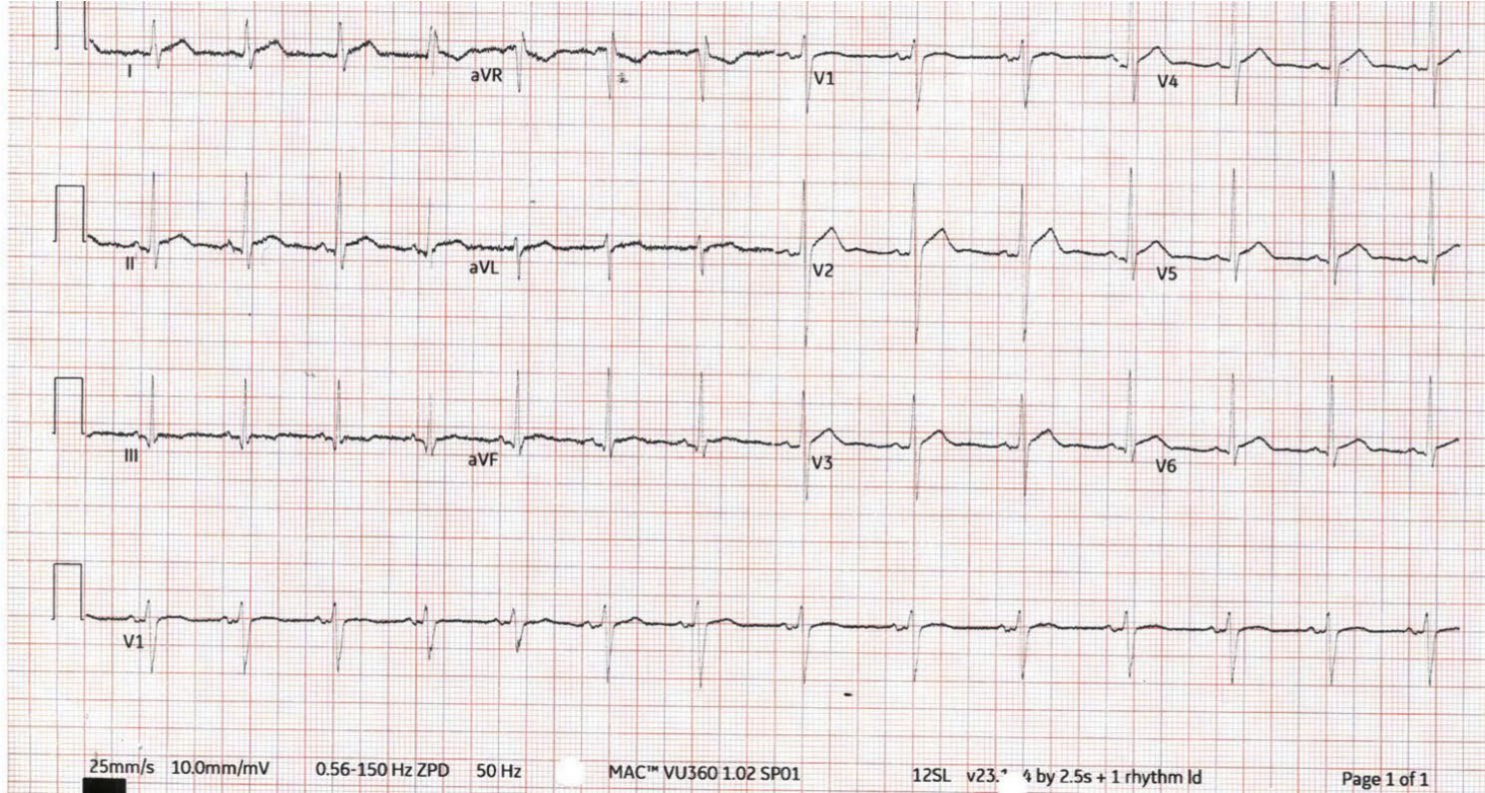
ECG on first presentation showing ST segment elevation most pronounced in V2, and PR depression, most pronounced lead II

**Fig. 2:**
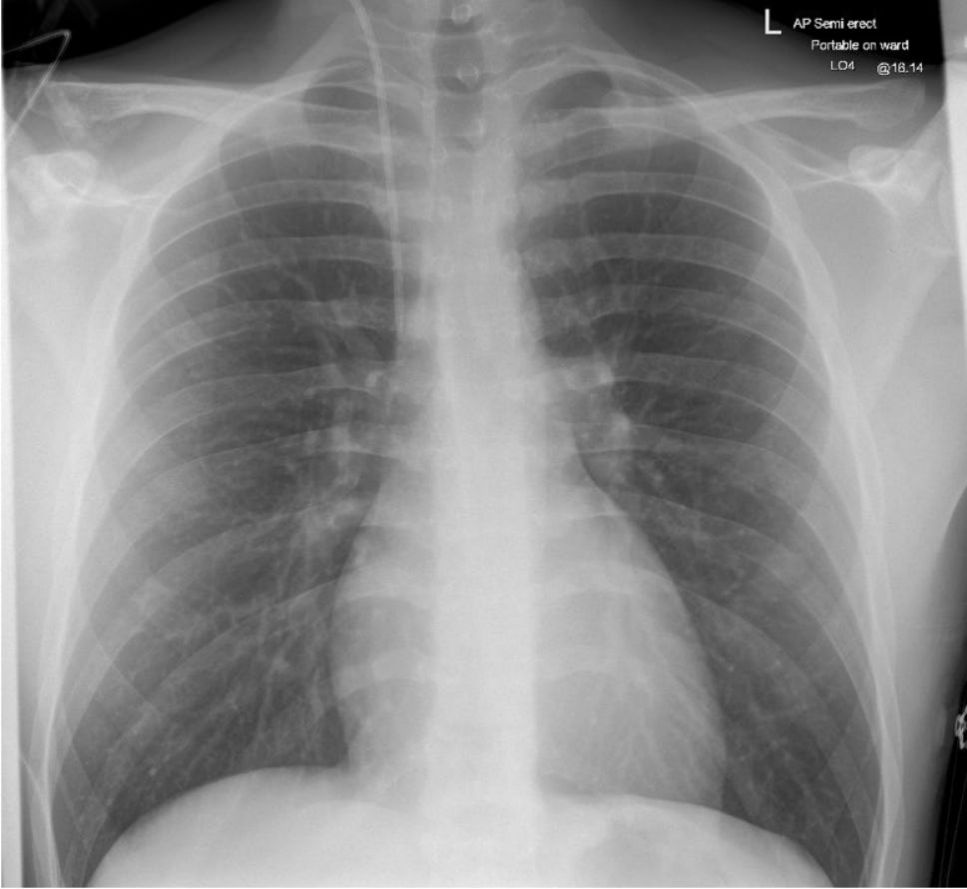
Chest x- ray on presentation showing normal lung fields

ECG on presentation revealed widespread saddle shaped ST elevation with PR depression (Fig. 1). Routine clinical observations, basic blood work including troponin and inflammatory markers, were unremarkable. Chest X-ray showed no focal effusion or consolidation (Fig. 2). A transthoracic echocardiogram (TTE) demonstrated a moderate, global pericardial effusion with no hemodynamic effect and no other abnormalities. A diagnosis of pericarditis was considered most likely, and he was treated with colchicine and non-steroidal anti-inflammatory drugs (NSAIDs) and discharged with plans for a follow up TTE in a few weeks’ time.

He was re-admitted a week later with worsening chest pain. ECG showed persistent saddle shaped ST elevation an PR depression. Bloods remained unremarkable with normal inflammatory markers and troponin. Repeat TTE showed a persistent, unchanged moderate pericardial effusion. He was discharged with a 3-month supply of colchicine. He had a further 3 emergency department (ED) attendances over the next few weeks with chest pain following which he was discharged with cardiology clinic follow up. He was followed up in the cardiology clinic five months after his initial presentation when his chest pain had resolved. ECG changes had returned to normal.

Given the repeated ED attendances, repeat TTE was requested which revealed a mass in the pericardial space adjacent to the RV free wall with an associated small effusion. The patient was referred for urgent cardiovascular magnetic resonance (CMR) at his local tertiary center to exclude malignancy.

**Fig. 3:**
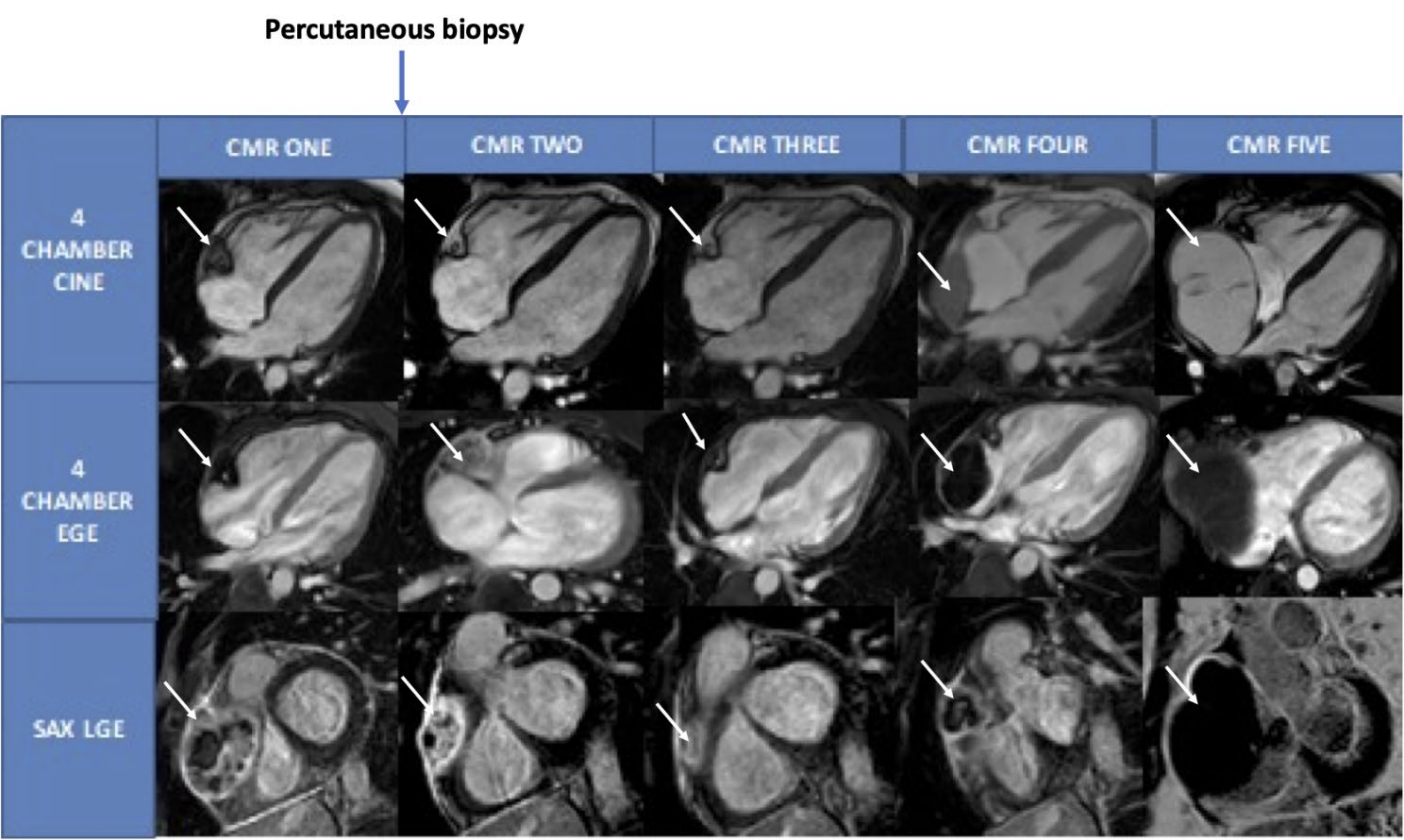
CMR images showing regression of cardiac angiosarcoma in CMR 2 and CMR 3 (post percutaneous biopsy) with subsequent increase in size of the mass in CMR 4. White arrow indicates cardiac tumor. EGE- early gadolinium enhancement; SAX- short axis; LGE- late gadolinium enhancement

CMR was undertaken 6 months after his initial admission with pericarditis. This showed normal biventricular size and function. There was a large, irregular heterogenous mass measuring 5.4 × 6.3 × 6.2 cm adjacent to the RA and RV extending from below the pulmonary bifurcation to the diaphragm (Fig. 3 (CMR 1), video 1). The mass compressed the basal RV free wall and the RA with suggestion of invasion into the mid RV free wall. The mass encompassed the right coronary artery (RCA) (Video 1). Mass tissue characterization revealed high signal on T1 and T2 weighted imaging and minimal change with fat suppression sequences with heterogenous tissue uptake on first pass contrast enhanced perfusion imaging. On early gadolinium enhancement (EGE), the mass was hypointense, and late gadolinium enhancement (LGE) revealed heterogenous enhancement with predominantly central necrosis (Fig. 3 (CMR 1), video 2). The heterogeneity of the mass, tissue characterization and vascularity were suggestive of a primary malignant cardiac tumor such as an angiosarcoma with alternative diagnoses including rhabdomyosarcoma, spindle cell sarcoma or a secondary deposit (renal, breast, melanoma or lung). Given rapid progression a CT thorax, abdomen and pelvis was undertaken to exclude a potential primary malignancy. CT did not reveal any lymphatic involvement or distant metastases.

The patient underwent percutaneous biopsy of the cardiac mass two weeks later without sternotomy. Biopsy findings were non-diagnostic, with a paucity of lesional cells obscured by fibrosis, inflammation and vasculature. No bacterial or fungal organisms were seen with Gram and PAS staining. Immunocytology was undertaken with negative staining of common antibodies and protooncogenes such as anaplastic lymphoma kinase (ALK-1), ROS-1 and human herpes virus-8 (HHV8). Serial CMR 3 weeks later showed significant reduction of the pericardial mass, disproportionate to the volume of tumor sampled (Fig. 3 (CMR 2), videos 3 and 4). PET-CT confirmed reduction in the size of the mass with moderate heterogeneous FDG uptake, more suggestive of malignancy rather than a benign lesion. CMR 6 weeks after baseline, was repeated to ensure the regression remained stable (Fig. 3 (CMR 3). Following suspected spontaneous regression of the mass, the case was discussed with local experts and a quaternary cardio-oncology center. All confirmed they had not seen a case of apparent spontaneous cardiac tumor regression of this magnitude in an adult. Differentials included immune-mediated processes such as giant cell myocarditis, immune-mediated regression of sarcoma, a percutaneous biopsy effect or a parasitic or fungal pseudotumor. CMR appearances were not suggestive of giant cell myocarditis and there was no histological evidence of parasitic or fungal pseudotumor.

**Fig. 4:**
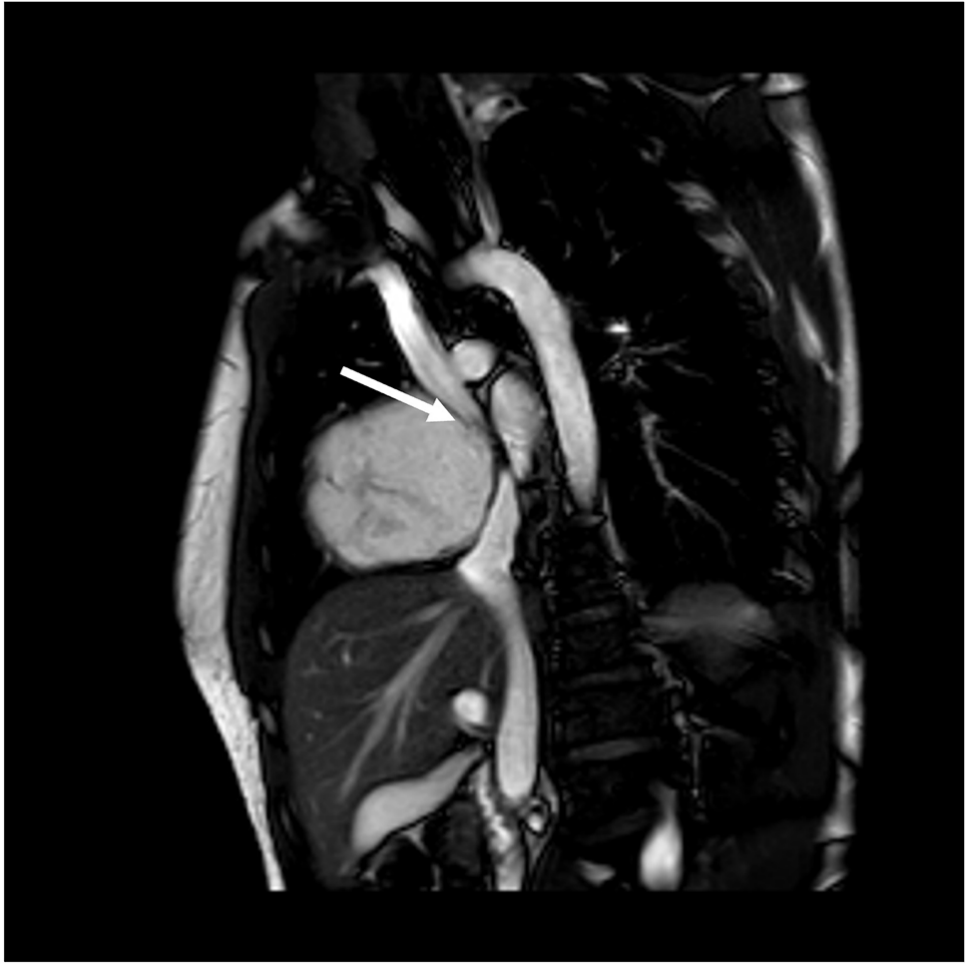
Superior vena cava (SVC) obstruction by cardiac angiosarcoma shown on CMR 5 (arrow)

**Fig. 5:**
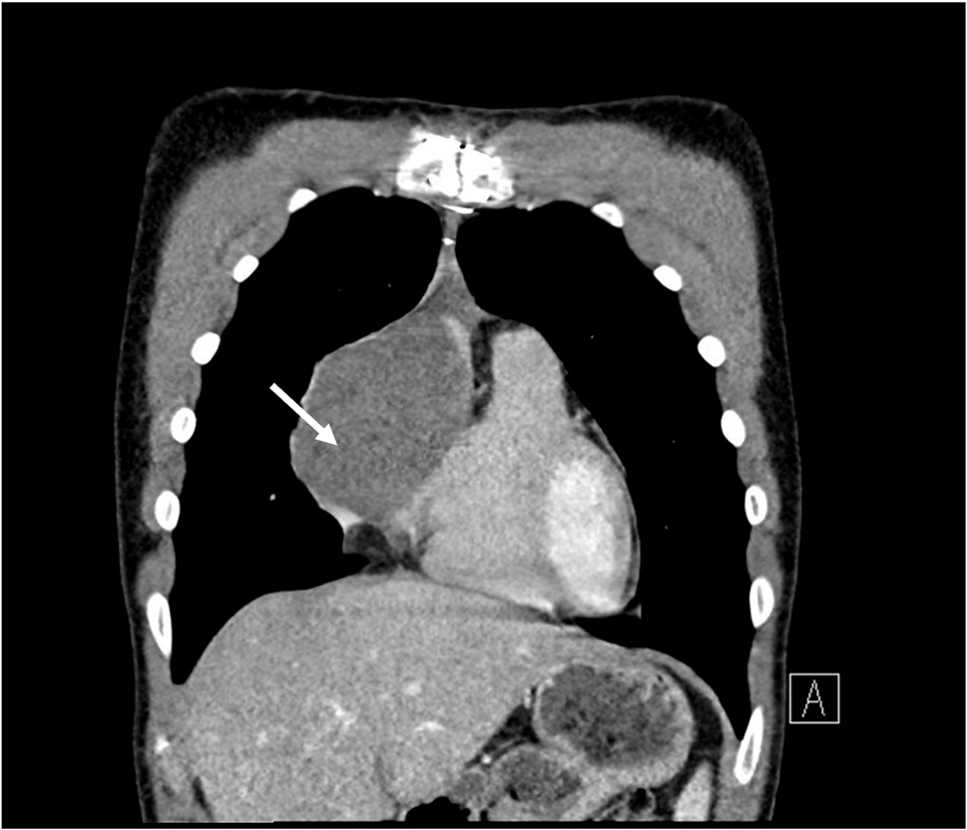
CT coronal slice confirming increase in size of the cardiac angiosarcoma (arrow)

Given the apparent resorption of the mass, repeat CMR was planned 3 months later. This scan (6 months after initial CMR) showed significant increase in the size of the cardiac mass (Fig. 3 (CMR 4)). Surgical biopsy confirmed AS and was considered surgically unresectable due to the extent of cardiac invasion. Post-biopsy, another CMR (30 weeks post baseline) was undertaken and showed persistent increase in the size of the angiosarcoma (Fig. 3 (CMR 5), video 5). The patient was started on paclitaxel as per the Angiotax trial data as well as targeted radiotherapy [4]. This failed to control the tumor, and he subsequently commenced a trial of doxorubicin, dexrazoxane and pazopanib. Recent CMR has shown a degree of superior vena cava (SVC) obstruction by the angiosarcoma (Fig. 4); the patient remained free of clinical signs or symptoms of SVC obstruction. CT confirmed the persistent increase in the size of the angiosarcoma (Fig. 5).

Further targeted radiotherapy has been undertaken with successful reduction in the size of the tumor. Given the positive response to radiotherapy, a surgical opinion was sought from the Royal Brompton Hospital, London. The patient very recently underwent complete surgical resection of the tumor, including resection of part of his right ventricle, resection of the right middle lobe of the lung (secondary to invasion of the tumor), a complete pericardiectomy and resection of the right phrenic nerve secondary to tumor involvement. His heart was reconstructed, and resection margins looked free of tumor macroscopically. He has made a good surgical recovery and at the time of publication has survived over 2 years from initial presentation.

## Discussions and conclusions

Spontaneous regression of cancer is defined as total or partial disappearance, without treatment or in the presence of known ineffective treatment in oncological diseases [5]. It is most commonly described in embryonal and breast cancer, renal adenocarcinoma, neuroblastoma, melanoma, sarcoma or carcinoma of the bladder or lymphoma [6]. This phenomenon is often associated with bacterial, fungal, viral or protozoan infection or vaccination therapy. There have been documented cases of spontaneous regression of cardiac rhabdomyoma in infants [7] but this does not extend to other primary cardiac tumors or adults.

The current case illustrates the significant reduction in size of an PCAS following percutaneous biopsy with subsequent significant increase in size of the mass 4 months later. It is conceivable that the initial regression may have been a percutaneous biopsy effect causing collapse or resorption of the necrotic core of the tumor [8]. Suggested mechanisms of how biopsy may lead to regression of tumors include disruption of the tumor feeding artery during biopsy or activation of the immune system [8]. This case illustrates that apparent regression of a mass cannot be considered as evidence against a malignant aetiology. Early diagnosis of PCAS remains challenging and the clinical manifestations of AS are atypical and can manifest as chest tightness, shortness of breath, arrythmia or symptoms of distal metastasis. Given the low incidence of AS, standard treatment guidelines do not exist, and treatment remains variable. PCAS has generally unfavorable prognosis with a variety of potential complications including constrictive pericarditis and metastatic disease [9–13]. However, successful remission has been reported with surgical resection and chemotherapy and/or radiotherapy [14, 15].

A recent case report describes the case of a 27-year-old man found to have PCAS with metastases to brain, lung and bone. Broncho-alveolar lavage and transbronchial biopsy showed atypical cells positive for Vimentin and CD3 highlighting the importance of immunohistochemistry in aiding the diagnosis [9]. Angiosarcoma, derived from endothelial cells, typically expresses endothelial markers (von Willebrand factor, cytokeratin, vimentin, CD31, CD34, and vascular endothelial growth factor (VEGF)). Another case report from John Hopkins described a difficult case of PCAS that initially presented with a right atrial pseudoaneurysm on CT [12]. Diagnosis was delayed by challenging histology in the presence of malignant cells initially obscured by extensive fibrin, granulation tissue and thrombus. Diagnosis was delayed until metastatic spread, at which point a cardio-phrenic metastatic lesion was biopsied. Histopathologically, angiosarcomas are difficult to diagnose due to cellular heterogeneity. This case report demonstrates the continued difficulty in diagnosis of AS and the need for multimodality and serial imaging as well as the importance of histopathology and immunohistochemistry.

## Conclusions

Cases of suspected primary cardiac malignancy require careful follow up with serial multimodality imaging [13]. Percutaneous biopsy effects should be considered in cases of tumor regression, and serial imaging should be planned afterwards.

### Electronic supplementary material

Below is the link to the electronic supplementary material.

**Supplementary Material 1: Video 1:** CMR 1. Left ventricular (LV) volume stack reveals a large cardiac mass attached to the right heart

**Supplementary Material 2: Video 2:** CMR 1. Late gadolinium enhancement (LGE), short axis (SAX) stack demonstrates a necrotic core and peripheral enhancement of the cardiac mass

**Supplementary Material 3: Video 3:** CMR 2. Left ventricular (LV) volume stack displays regression of the cardiac mass compared to CMR 1

**Supplementary Material 4: Video 4:** CMR 2. Late gadolinium enhancement (LGE) short axis (SAX) stack shows regression of the cardiac mass and necrotic core compared to CMR 1

**Supplementary Material 5: Video 5:** CMR 5. Right ventricular (RV) volume stack shows significant progression of the cardiac angiosarcoma

## Data Availability

No datasets were generated or analysed during the current study.

## Abbreviations

PCAS: Primary Cardiac Angiosarcoma
CMR: Cardiovascular magnetic resonance
ED: Emergency department
SVC: Superior vena cava
TTE: Transthoracic echocardiography
RA: Right atrium
RCA: Right coronary artery
RV: Right ventricle
LGE: Late gadolinium enhancement
